# Natural Killer Cells Are Dispensable for Virus Control in *Rag2^−/−^* Mice During Primary RSV Infection

**DOI:** 10.1002/eji.70045

**Published:** 2025-09-03

**Authors:** Joy Nakawesi, Tammie Sow Tao Min, Cecilia Johansson

**Affiliations:** ^1^ Respiratory Infections Section National Heart and Lung Institute, Imperial College London London UK

**Keywords:** IFN‐γ, NK cells, Rag2^−/−^ mice, respiratory syncytial virus

## Abstract

Respiratory syncytial virus (RSV) is one of the major causes of severe lower respiratory tract infections, especially in children, the elderly, and immunocompromised individuals. Complications arising from viral infections in these age groups can present therapeutic challenges, as most of these individuals have impaired adaptive immunity. Using the T‐ and B cell‐deficient *Rag2^−/−^* mice, the mechanisms that mediate protection in immunocompromised hosts during RSV infection can be investigated. RSV‐infected *Rag2^−/−^* mice showed no symptoms of disease or chronic inflammation in the lungs and airways despite the presence of infectious virus in their lungs several months after infection. Interestingly, Natural Killer (NK) cells, the main innate cells with anti‐viral cytotoxic effector functions, were recruited 2 days earlier in the lungs of *Rag2^−/−^* mice compared with wildtype mice, resulting in early production of IFN‐γ. However, depletion of NK cells did not affect disease severity or viral load. Together, these results suggest that the NK cells are largely dispensable for virus control during primary RSV infection in *Rag2^−/−^* mice.

## Graphical abstract

*Rag2^−/−^* mice are unable to clear respiratory syncytial virus (RSV) infection, despite an earlier recruitment of natural killer (NK) cells to the lungs compared with wild‐type controls. Furthermore, depletion of NK cells does not affect viral load, indicating that alternative innate anti‐viral mechanisms may compensate in the absence of adaptive immunity.

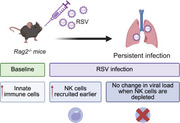

## 1 Introduction

Respiratory syncytial virus (RSV) is the most common cause of acute lower respiratory tract infections in children [1]. Globally, RSV accounts for approximately 33 million acute lower respiratory infection episodes, 3.6 million hospital admissions, and 26,300 in‐hospital deaths in children below 5 years. In healthy adults, RSV manifests as a mild illness. However, RSV infections cause significant morbidity and mortality in the elderly, especially those with pre‐existing conditions, including immunocompromised individuals [2, 3]. RSV infection does not result in long‐lasting immunity, and thus reinfections are common [4]. Despite continued efforts for several decades, the current treatments for RSV are predominantly supportive care [4]. For prevention, monoclonal antibodies specific to the RSV fusion protein can be given to high‐risk infants, and recently, RSV vaccines were licensed for use in adults above 60 years [5, 6], or in pregnant women (to prevent RSV infections in infants) [7, 8]. However, there is still a lot to uncover about the immune mechanisms driving disease severity during RSV infection.

RSV is a single‐stranded, negative‐sense RNA virus [9]. Upon infection of the host, RSV is recognised by pattern recognition receptors (PRRs) expressed on lung‐resident cells, such as epithelial cells and alveolar macrophages (AMs), that initiate the antiviral innate immune response by secreting chemokines and cytokines such as type I interferons (IFNs) [9, 10]. We have previously shown that AMs are the main producers of the initial type I IFNs in the lung during RSV infection [11]. Type I IFNs play a key role in limiting viral replication [12] but also regulate immune responses by promoting chemokines and cytokines that result in the recruitment and activation of immune cells, respectively [13, 14]. Neutrophils are the first cells to be recruited to the lungs during RSV infection [15, 16, 17, 18] by chemokines such as CXCL1 and CXCL2, which are detectable in the lungs as early as 4 to 8 h after virus exposure [12, 16, 18]. The next cell type to infiltrate the lung is the monocytes, which peak at day 2 post‐infection [11]. Type I IFNs are key in recruiting inflammatory monocytes by inducing the production of monocyte chemoattractants such as CCL2 [11]. Natural killer (NK) cells are also recruited to the lungs, peaking at day 4 post‐infection [10]. A study by Wang et al. [19] showed that the lungs have the highest percentage of NK cells, which also maintained a more mature phenotype, compared with other peripheral tissues. Furthermore, macrophages can enhance NK cell recruitment and activation via their early production of type I IFNs [20]. NK cells have been implicated in early defense against several respiratory viral infections by their production of anti‐viral cytokines, such as IFN‐gamma (IFN‐γ), which can amplify the inflammatory response, and their secretion of granzymes and perforins, which directly kill infected cells [21, 22, 23]. For example, during respiratory vaccinia virus infection, NK cells were shown to be the primary source of IFN‐γ before the arrival of CD8^+^ T cells [24]. On the other hand, NK cell activity has been implicated in exacerbating pathology, promoting lung injury and mortality at the early stages of RSV [25] and influenza virus [26, 27] infection in mice.

Adaptive immune responses are indispensable for viral clearance and protection from re‐ infection [10]. RSV‐specific antibodies are generated after infection, and they are usually very effective in neutralising the virus [28, 29, 30]. However, they are poorly maintained and decline to pre‐infection levels within a few months of infection, making them insufficient to prevent re‐infection with RSV long‐term [10, 31]. T cells are also crucial in the defense against RSV, especially CD8^+^ T cells, which have an important role in the clearance of the primary infection and limiting infection during secondary RSV infection [10, 32, 33, 34, 35].

In immunocompromised individuals, in whom the adaptive immune responses are impaired, it is not completely understood how the innate immune responses control RSV infection. In mouse models lacking functional T cells, complete viral clearance has been shown during influenza virus infection [36], while higher viral titres and increased airway inflammation were shown during RSV infection [37]. While the mechanisms that mediate protection in immunocompromised hosts are still unclear, in this study, we used the T‐ and B‐cell‐deficient *Rag2^−/−^* mice to investigate the innate immune responses that can mediate protection in the absence of adaptive immunity during primary RSV infection. We found that *Rag2^−/−^* mice were not susceptible to primary RSV infection but failed to fully clear the virus from their lungs and showed a long‐term infection. *Rag2^−/−^* mice also showed an increased number of innate immune cells present in the lungs before infection. Interestingly, NK cells, which are the main innate immune cells with cytolytic activity, were recruited 2 days earlier in the lungs of *Rag2^−/−^* mice compared with wildtype mice, resulting in the early production of IFN‐γ. However, depletion of NK cells did not affect disease severity or viral load, indicating that other innate anti‐viral mechanisms are controlling RSV in immunocompromised *Rag2^−/−^* hosts.

## 2 Results

### 2.1 *Rag2* ^−/−^ Mice Are Not Susceptible to Primary RSV Infection Despite Sustained Viral Load

**FIGURE 1:**
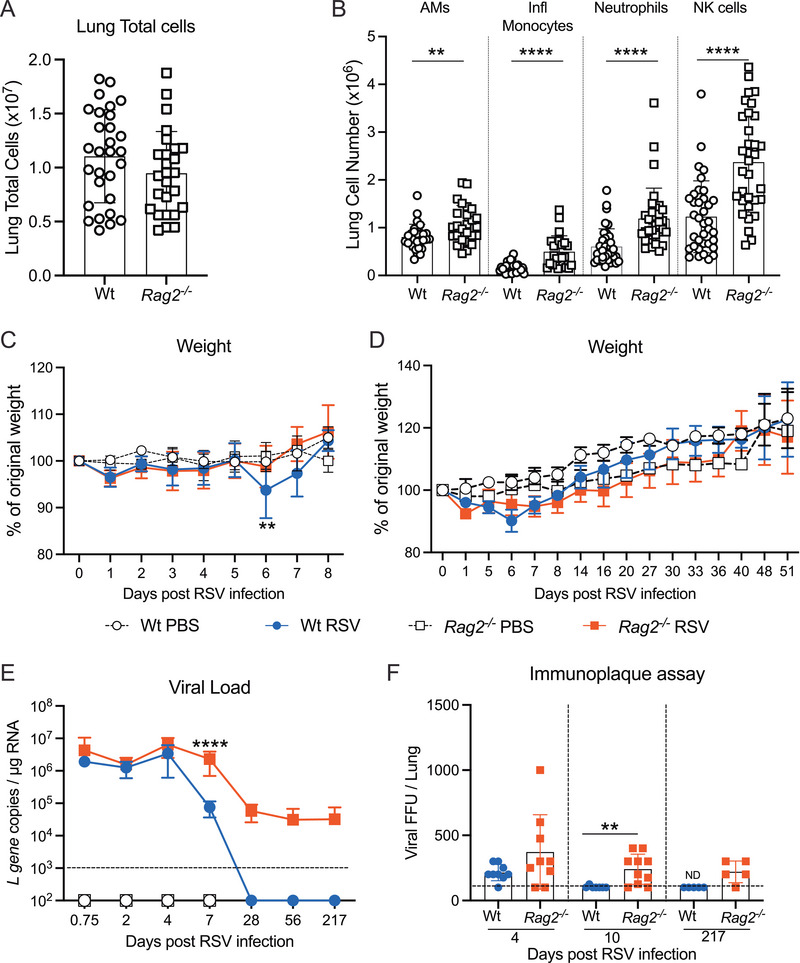
**Uninfected *Rag2^−^^/^^−^* mice exhibit a higher number of innate immune cells in the lungs compared with Wt mice, yet they display a sustained viral load after primary RSV infection**. Lung cells from uninfected Wt and *Rag2^−/−^* mice were analysed. (**A**) The total number of cells in the lungs. (**B**) The total number of alveolar macrophages (AMs; CD45^+^SiglecF^+^CD11c^+^), inflammatory monocytes (CD45^+^CD11b^+^CD64^+^), neutrophils (CD45^+^Ly6G^+^SiglecF^−^), and NK cells (CD45^+^CD49b^+^) enumerated in the lungs of uninfected mice. Wt and *Rag2^−/−^* mice were mock (PBS) or RSV‐infected i.n. and the lungs were analysed at different time points after infection. (**C, D**) Weight loss is shown as the percentage of the original weight. (**E**) Lung viral load determined by RT‐qPCR quantification of RSV *L* gene expression in lung tissue. (**F**) Infectious viral titer (focus‐forming units) was measured in the lungs by immunoplaque assay. The detection limit is shown as a dotted line. Data are presented as the mean ± SEM of (**A, B**) *n* = 29 Wt and *n* = 26 *Rag2^−/−^* mice pooled from seven independent experiments, (**C**) *n* = 8–10 mice/group pooled from two independent experiments, (**D**) *n* = 3–6 mice from one experiment representative of two independent experiments. (**E**) Data are presented as the mean ± SEM of *n* = 6–10 mice/group pooled from two independent experiments (day 0.75, 4, 7, and 56), *n* = 10–13 mice/group pooled from three independent experiments (day 2), and *n* = 3–5 mice/group from one experiment (day 28 and 217). PBS control groups are only shown until day 7. (**F**) Data are presented as the mean ± SEM *n* = 4–5 Wt or *Rag2^−/−^* (day 4) and *n* = 3–4 Wt and *n* = 10 *Rag2^−/−^* (day 10) mice pooled from two independent experiments and *n* = 5 Wt or *Rag2^−/−^* (day 217) mice from one experiment. Each symbol represents an individual mouse. Statistical significance was determined by (A, B, and F). Student's *t*‐test, (C–E) one‐way ANOVA with Tukey's post hoc test per time point, and only significance between RSV groups is shown. **p = 0.01, ****p = 0.0001. Symbols: open circle: Wt PBS; open square: *Rag2^−/−^* PBS; blue circle: Wt RSV; red square: *Rag2^−/−^* RSV.

As a few innate immune cells are always present in the lungs, we sought to first characterise the innate immune cells present in the lungs of uninfected C57BL/6 wildtype (Wt) or recombinant activation gene 2 knockout (*Rag2^−/−^*) mice. Both Wt and *Rag2^−/−^* mice had similar numbers of total lung cells (**Figure** **1A**). Consistent with previous studies [37, 38, 39], we observed that uninfected *Rag2^−/−^* mice had significantly more AMs, inflammatory monocytes, neutrophils, and NK cells in their lungs compared with Wt mice (**Figure** **1B** **;** for gating strategy, see **Figure** **S1**). Rarer cell types such as innate lymphoid cells 1 and 2 (ILC1 and ILC2) were also increased in the lungs of uninfected *Rag2^−/−^* mice compared with Wt mice (**Figure** **S2**).

Since the anti‐viral innate immune responses start immediately upon viral infections, we investigated the role of this response in the absence of a functional adaptive immune response during primary RSV infection. Wt and *Rag2^−/−^* mice were intranasally infected with RSV, and disease severity, measured by weight loss, and viral load were monitored. As expected, RSV‐infected Wt mice lost about 7% of their original weight on day 6 post‐infection (p.i.) and recovered by day 8 p.i. while no weight loss was observed in the *Rag2^−/−^* mice or the PBS controls (**Figure** **1C**). In addition, none of the RSV‐infected mice lost weight later during the infection (**Figure** **1D**). Despite the lack of weight loss, RSV‐infected *Rag2^−/−^* mice had significantly higher RSV *L* gene copy numbers as quantified by RT‐qPCR on day 7 p.i. The RSV *L* gene was also detected in the *Rag2^−/−^* mice at 28, 56, and 217 days p.i. while the Wt mice had cleared the virus at these time points (**Figure** **1E**). By using the RSV immunoplaque assay, it was confirmed that the infected *Rag2^−/−^* mice were harboring infectious virus for much longer than Wt mice, as live virus was still detectable 7 months p.i. (**Figure** **1F**). Together, these results show that *Rag2^−/−^* mice do not develop symptoms or signs of RSV infection despite harbouring more virus and failing to fully clear the virus from their lungs.

### 2.2 Kinetics of Innate Immune Cell Infiltration into the Lungs During RSV Infection in *Rag2* ^−/−^ Mice

**FIGURE 2:**
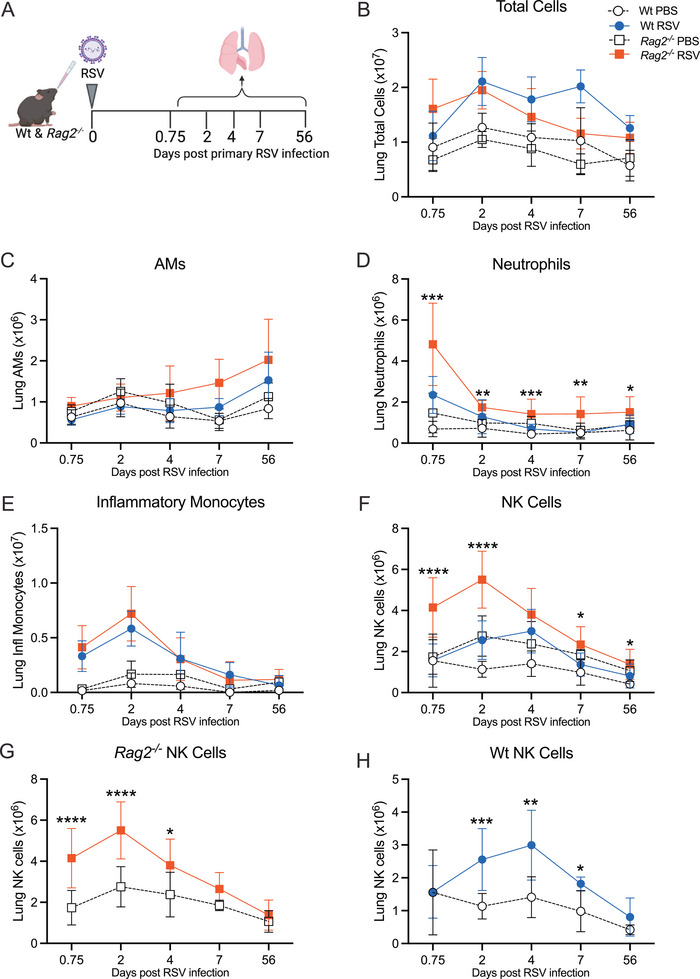
**Innate immune cells in the lungs after RSV infection of *Rag2^−^^/^^−^* and Wt mice**. (A) Wt and *Rag2^−/−^* mice were mock (PBS) or RSV‐infected i.n. and cells from the lungs were analysed at different time points after infection. (**B**) Total cells, (**C**) AMs, (**D**) neutrophils, (**E**) inflammatory monocytes, and (**F**) NK cells in the lungs, (**G, H**) NK cell data from (**F**) showing *Rag2^−/−^* and Wt mice separately. Data are presented as the mean ± SEM of *n* = 7–9 mice/group pooled from two independent experiments (day 0.75), *n* = 10–12 for PBS groups and *n* = 22 for RSV‐infected mice pooled from three or five independent experiments respectively (day 2), *n* = 7–10 for PBS groups and *n* = 14–16 for RSV‐infected mice pooled from 2–3 or 3–4 independent experiments, respectively (day 4), *n* = 3 for PBS groups (from one experiment) and *n* = 9–10 for RSV‐infected mice pooled from two independent experiments (day 7), and *n* = 3–6 for PBS groups and *n* = 7–10 for RSV‐infected mice pooled from 1–2 or 2 independent experiments respectively (day 56). Statistical significance was determined by one‐way ANOVA with Tukey's post hoc test per time point, and only significance between RSV groups is shown. *p < 0.05, **p < 0.01, ***p < 0.001, ****p < 0.0001. Symbols: open circle: Wt PBS; open square: *Rag2^−/‐^* PBS; blue circle: Wt RSV; red square: *Rag2^−/−^* RSV.

Next, the kinetics of innate immune cell recruitment/infiltration into the lungs in both Wt and *Rag2^−/−^* mice were investigated during RSV infection (**Figure** **2A**). Both RSV‐infected Wt and *Rag2^−/−^* mice had similar numbers of total cells and AMs in the lungs throughout the infection (**Figure** **2B**,**C**). Neutrophils are the first cells to be recruited into the lungs during RSV infection [16, 17]. As expected, their numbers peaked in the lungs at 18 h p.i. returning to baseline levels by day 4 p.i. RSV‐infected *Rag2^−/−^* mice had significantly more neutrophils compared with the RSV‐infected Wt mice throughout the infection (**Figure** **2D**). Inflammatory monocytes were recruited into the lungs of both RSV‐infected Wt and *Rag2^−/−^* mice by 18 h p.i. peaking at day 2 p.i. and returning to baseline levels by day 7 p.i. (**Figure** **2E**). The NK cell numbers were significantly higher in the RSV‐infected *Rag2^−/−^* mice compared with the RSV‐infected Wt mice throughout the infection. NK cells were recruited into the lungs of RSV‐infected *Rag2^−/−^* mice earlier, at 18 h p.i. peaking at day 2 p.i. and returning to baseline levels by day 7 p.i. (**Figure** **2F**,**G**). On the other hand, NK cells were recruited into the lungs of RSV‐infected Wt mice at day 2 p.i., peaked at day 4 p.i., and returned to baseline levels by day 7 p.i. (**Figure** **2F**,**H**). Interestingly, the PBS‐*Rag2^−/−^* mice had a higher baseline of NK cells, at similar numbers to the NK cell numbers in the RSV‐infected Wt mice (**Figure** **2F**). Altogether, these results show that innate immune cells are recruited into the lungs of both Wt and *Rag2^−/−^* mice during primary RSV infection, albeit the significantly higher numbers of neutrophils and NK cells recruited to the lungs of RSV‐infected *Rag2^−/−^* mice. Despite the sustained viral load, *Rag2^−/−^* mice had no signs of chronic inflammation in the lungs (**Figure** **2**) or airways (**Figure** **S3**).

### 2.3 Early IFN‐γ Production in *Rag2* ^−/−^ Mice During Primary RSV Infection

**FIGURE 3:**
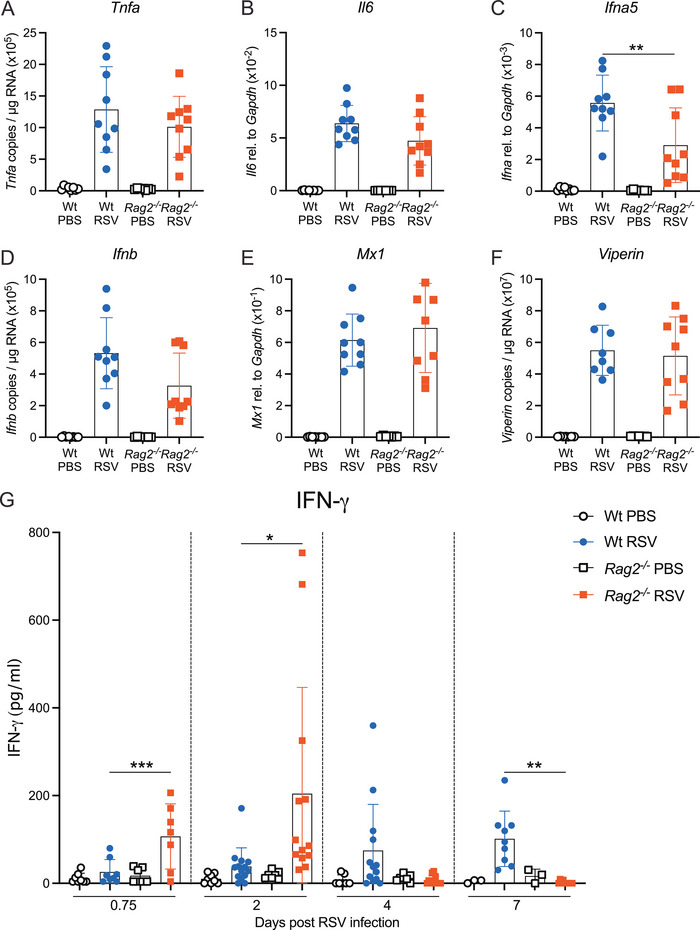
**RSV Infection induces IFN‐γ earlier in the lungs of *Rag2^−^^/^^−^* mice compared with Wt mice**. Wt and *Rag2^−/−^* mice were mock (PBS) or RSV‐infected i.n. and the lungs were analysed at 18 h (day 0.75) p.i. mRNA expression of (**A**) *Tnfa*, (**B**) *Il6*, (**C**) *Ifna5*, (**D**) *Ifnb*, (**E**) *Mx1*, and (**F**) *Viperin* was quantified by RT‐qPCR in the lung tissue. (**G**) IFN‐γ levels in the bronchoalveolar lavage fluid were analysed by ELISA at day 0.75, 2, 4, and 7 p.i. Data are presented as the mean ± SEM of (**A**–**F**) *n *= 7–9 mice/group, pooled from two independent experiments. (**G**) *n *= 7–8 mice/group pooled from two independent experiments (day 0.75), *n *= 7–8 for PBS groups and *n *= 12–14 for RSV‐infected mice pooled from 2 or 3 independent experiments respectively (day 2 and 4) and *n *= 3 for PBS groups (from one experiment) and *n *= 9–10 for RSV‐infected mice pooled from two independent experiments (day 7). Each symbol represents an individual mouse. Statistical significance was determined by one‐way ANOVA with Tukey's post hoc test per time point, and only significance between RSV groups is shown. *p < 0.05, **p < 0.01, ***p < 0.001. Symbols: open circle: Wt PBS; open square: *Rag2^−/−^* PBS; blue circle: Wt RSV; red square: *Rag2^−/−^* RSV.

We assessed the expression of different cytokines, chemokines, and IFN‐stimulated genes (ISGs) by RT‐qPCR in the lungs of both RSV‐infected Wt and *Rag2^−/−^* mice at 18 h p.i. The expression of *Tnfa* and *Il6* was similar in both the Wt and *Rag2^−/−^* infected mice (**Figure** **3A**,**B**). *Ifna5* expression was significantly higher in the Wt compared with the *Rag2^−/−^* mice (**Figure** **3C**), but *Ifnb* and the ISGs, *Mx1* and *Viperin*, were expressed to the same levels in the lungs of Wt and *Rag2^−/−^* infected mice (**Figure** **3D**–**F**). Since *Rag2^−/−^* mice lack a functional mature adaptive immune system, NK cells are the major cells present in these mice that are capable of secreting IFN‐γ [40]. Therefore, IFN‐γ levels were assessed in the BAL (airways) in both the Wt and *Rag2^−/−^* mice by ELISA. In line with our observations that NK cells are recruited already at 18 h p.i. in RSV‐infected *Rag2^−/−^* mice (**Figure** **2F**), they also showed more IFN‐γ at 18 h p.i. (**Figure** **3G**). Moreover, the IFN‐γ response peaked at day 2 p.i. and returned to baseline levels by day 4 p.i. while in RSV‐infected Wt mice, IFN‐γ was only detectable at days 4 and 7 p.i. (**Figure** **3G**) directly coinciding with NK and T cell recruitment into the lungs (**Figure** **2H**; **Figure** **S4**). Interestingly, the granzyme B levels in the BAL were similar in both groups of mice until day 7 p.i. when the RSV‐infected Wt mice had a trend to more granzyme B compared with the RSV‐infected *Rag2^−/−^* mice (**Figure** **S5**). Together, these results show that the early NK cell recruitment and IFN‐γ production in the lungs could account for the innate anti‐viral mechanisms in the *Rag2^−/−^* mice during primary RSV infection.

### 2.4 Cytokine Stimulation Promotes NK Cell Effector Functions, but NK Cells Are Dispensable for Viral Control During Primary RSV Infection

**FIGURE 4:**
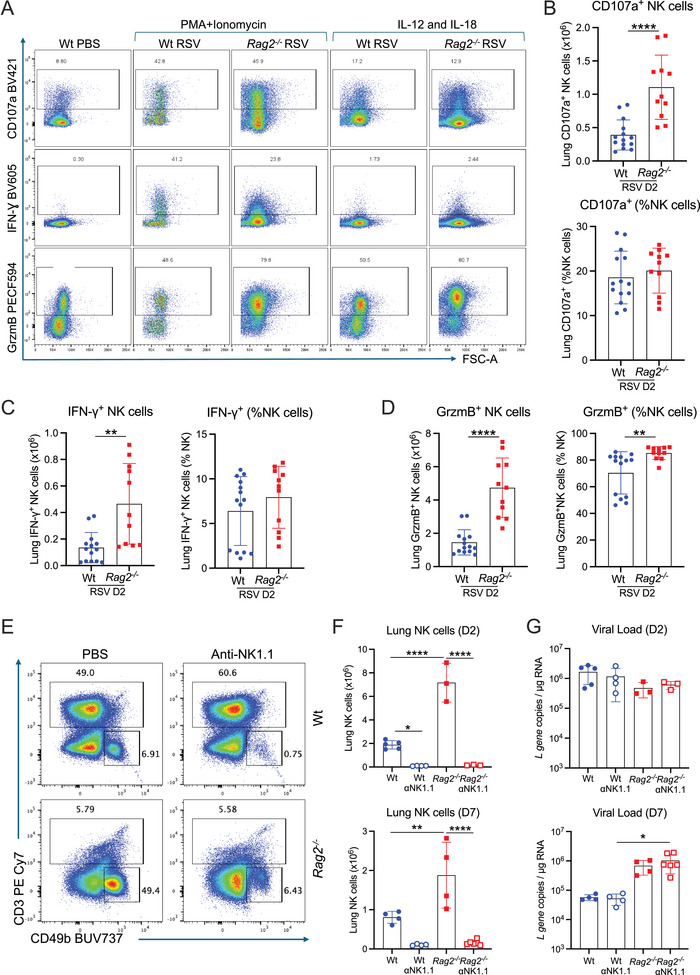
**Depletion of NK cells does not alter viral clearance in *Rag2^−^^/^^−^* mice during RSV infection**. Wt and *Rag2^−/−^* mice were RSV‐infected i.n. On day 2 p.i., lung cells were stimulated ex vivo with IL‐12 and IL‐18 or PMA and ionomycin as a positive control. The cells were subsequently stained for surface CD107a, intracellular IFN‐γ, and granzyme B. NK cells in the lungs were analysed using flow cytometry. (**A**) Representative flow cytometry plots showing CD107a, IFN‐γ, and granzyme B on or in CD49b^+^ NK cells (for gating strategy see Figure S1) from Wt or *Rag2* ^−/−^ mice after PBS, PMA+Ionomycin, or IL‐12/IL‐18 stimulation. Lung cells from RSV‐infected Wt or *Rag2* ^−/−^ mice restimulated with IL‐12/IL‐18, and the total number and frequency of (**B**) CD107a^+^, (**C**) IFN‐γ^+^, and (**D**) Granzyme B^+^ NK cells were determined. (**E**) Representative flow cytometry plots of T and NK cells in the lungs of Wt or *Rag2* ^−/−^ mice with or without NK cell depletion on day 2 p.i. (**F**) The number of NK cells in the lungs of Wt or *Rag2* ^−/−^ mice after NK cell depletion on days 2 and 7 p.i. (**G**) Lung viral load determined by RT‐qPCR quantification of RSV *L* gene expression in lung tissue on days 2 and 7 p.i. Data are presented as the mean ± SEM of (**B–D**) Wt *n* = 14 mice and *Rag2^−/−^ n* = 11 mice pooled from two independent experiments. (**E**–**G**) Wt *n* = 4–5 and *Rag2^−/−^ n* = 3 mice from one experiment, representative of two experiments (day 2), and Wt *n* = 4 mice and *Rag2^−/−^ n* = 4–5 mice from one experiment (day 7). Each symbol represents an individual mouse. Statistical significance was determined by (B–D) Student's *t*‐test and (F, G) one‐way ANOVA with Tukey's post hoc test. *p < 0.05, **p < 0.01, ****p < 0.0001. Blue symbols: Wt RSV; red symbols: *Rag2^−/−^* RSV.

Cytokines such as IL‐12 and IL‐18 can directly activate and induce NK cells to produce IFN‐γ and degranulate and release cytolytic effector molecules like granzymes [19, 41, 42]. To assess the effector functions of NK cells, lung cells were stimulated *ex vivo* with recombinant IL‐12 and IL‐18 for 4 h. Restimulation of NK cells from the lungs of uninfected *Rag2^−/−^* or Wt mice showed similar granzyme B and IFN‐γ induction (**Figure** **S6**), indicating that the ability of NK cells from a *Rag2^−/−^* mouse to produce cytokines is not different to NK cells from a Wt mouse. Following up on our observations that the IFN‐γ response peaked at day 2 p.i. and returned to baseline levels by day 4 p.i. in the RSV‐infected *Rag2^−/−^* mice (**Figure** **3G**), recombinant IL‐12 and IL‐18 *ex vivo* stimulation of lung cells after RSV infection showed that indeed the number of NK cells producing intracellular IFN‐γ and undergoing degranulation (as measured by surface expression of CD107a) peaked on day 2 p.i. and were decreased by day 4 p.i. in the RSV‐infected *Rag2^−/−^* mice (**Figure** **S7**). We thus analysed the NK cell effector functions in both the RSV‐infected Wt and *Rag2^−/−^* mice on day 2 p.i. We observed that the number of NK cells that showed degranulation was significantly higher in *Rag2^−/−^* mice compared with Wt mice (**Figure** **4A**,**B** **;** **Figure** **S7**). As anticipated, the number of NK cells producing intracellular IFN‐γ and granzyme B (GrzmB) was higher in RSV‐infected *Rag2^−/−^* mice compared with RSV‐infected Wt mice (**Figure** **4A**,**C**,**D**). However, the frequency of CD107a^+^ NK cells were similar or lower in RSV‐infected *Rag2^−/−^* mice compared with Wt mice (**Figure** **4B** **;** **Figure** **S7**), and the frequency of IFN‐γ producing NK cells were similar in RSV‐infected *Rag2^−/−^* mice compared with Wt mice (**Figure** **4C** **;** **Figure** **S7**), while the frequency of GrzmB producing NK cells were higher in RSV‐infected *Rag2^−/−^* mice compared with Wt mice (**Figure** **4D**). Altogether, these data suggest that NK cells in the lungs of both Wt and *Rag2^−/−^* mice are similarly activated to degranulate and to produce IFN‐γ and granzyme B. However, because *Rag2^−/−^* mice have a higher number of NK cells, their overall effect may be more pronounced.

Next, we assessed whether the earlier emergence of NK cell effector activity in RSV‐infected *Rag2^−/−^* mice contributed to virus control in the lungs during primary RSV infection. NK cells were depleted in both Wt and *Rag2^−/−^* mice using an anti‐NK1.1 antibody (**Figure** **4E**,**F**). After RSV infection, Wt mice, but not *Rag2^−/−^* mice, lose weight (**Figure** **1C** **;** **Figure** **S8A**). Interestingly, despite no detectable gene expression of IFN‐γ in the lungs of NK cell‐depleted *Rag2^−/−^* mice (**Figure** **S8B**), there were no differences in weight loss (**Figure** **S8A**) or RSV *L* gene copy numbers as quantified by RT‐qPCR on days 2 and 7 p.i. between the NK cell‐depleted and non‐depleted Wt and *Rag2^−/−^* RSV‐infected mice (**Figure** **4G**). Altogether, these results indicate that although NK cells are recruited earlier to the lungs, they are dispensable for virus control during primary RSV infection in *Rag2^−/−^* mice.

## 3 Discussion

During viral infections, adaptive immunity is indispensable for viral clearance and protection from reinfection in mice [33, 43, 44]. Furthermore, immunocompromised people are more susceptible to respiratory infections, especially if they are lymphopenic [45, 46, 47]. In the present study, T‐ and B‐cell‐deficient *Rag2^−/−^* mice were used to investigate how RSV infection is altered in the absence of adaptive immunity. Consistent with previous studies [37, 38, 39], we found that uninfected *Rag2^−/−^* mice had significantly more innate immune cells in their lungs compared with Wt mice. Overall, the innate immune response in RSV‐infected Wt and *Rag2^−/−^* mice was similar, except that NK cells infiltrated the lungs earlier in *Rag2^−/−^* mice. This suggests that *Rag2* ^−/−^ mice may compensate for the absence of mature T‐ and B cells through early NK cell infiltration. However, this did not influence early viral load during RSV infection. Surprisingly, RSV‐infected *Rag2* ^−/−^ mice harboured live infectious virus months after infection. Notably, despite this long‐term sustained viral load, *Rag2^−/−^* mice exhibited no symptoms of disease (measured by weight loss or clinical signs) and showed no chronic inflammation in the lungs or airways.

There are many players involved in the control of lung viral infections. Type I IFNs, monocytes, NK cells, and T cells are key host mechanisms that control virus replication and spread [10, 13]. After RSV‐infection, the levels of type I IFNs, ISGs, monocytes, and neutrophils were very similar in *Rag2^−/−^* mice compared with Wt mice, and a difference in viral load was not observed until day 7 p.i. However, NK cells were recruited into the lungs of *Rag2^−/−^* mice as early as day 2 p.i., leading to an early production of IFN‐γ. In line with our findings, Xiong et al. [37] showed that *Rag2^−/^* mice had similar viral load as Wt mice until day 11 p.i. and then failed to fully clear the infection even though the mice had increased NK cells in peripheral blood. Furthermore, RSV‐infected nude mice (lacking mature T cells) also showed increased viral load and airway inflammation compared with Wt BALB/c mice [34]. Indeed, since NK cells are the main innate immune cell population with anti‐viral cytotoxic effector functions present in *Rag2^−/−^* mice [21, 22, 23, 45], we reasoned that they might be the major drivers of viral control during primary RSV infection. To our surprise, this was not the case, as the NK cell‐depleted, RSV‐infected *Rag2^−/−^* mice showed no differences in viral load or disease severity as measured by weight loss.

Exploring the effect of NK cell depletion in other lung viral infections has reported different outcomes. One study using the respiratory vaccinia virus infection model showed that depletion of NK cells and/or IFN‐γ in *Rag2^−/−^* mice significantly accelerated clinical manifestations, and mice succumbed more rapidly to vaccinia virus infection than the non‐NK cell‐depleted mice [24]. In contrast, when NK cells were depleted during influenza virus infection, one study showed significantly impaired viral clearance [48], while another study reported no effect on lung viral load [27]. However, several studies have reported less pathology in the lungs after influenza virus infection in NK cell‐depleted mice [26, 27], suggesting that NK cells can exacerbate pathology and promote mortality during influenza virus infection [26, 27].

Although NK cells seem to play a dual role during viral infections, both aiding in viral control and contributing to immunopathology [23], our study suggests that NK cells and innate IFN‐γ are not essential for limiting viral replication during RSV infection. While we do not establish a definitive role for NK cells in controlling disease severity, it remains unclear how their early recruitment and IFN‐γ production in the lungs of *Rag2^−/−^* mice affect RSV infection. We believe that NK cells in *Rag2* ^−/−^ mice are fully functional. However, further investigation into their phenotype and their *in situ* location relative to the virus in the lungs at later time points would be valuable. Notably, Reilly et al. [49] reported elevated NK cells in the airways of children with severe RSV infection. In these children, circulating NK cells were decreased, and they exhibited impaired cytotoxicity [49], indicating that this altered NK cell phenotype is associated with severe RSV disease in infants. Taken together, these findings suggest that the severity of infection may influence the NK cell phenotype and their role in both viral clearance and immunopathology.

Interestingly, *Rag2* ^−/−^ mice do not show any weight loss at any time after primary RSV infection, even though the virus is not completely cleared from their lungs. Weight loss is dependent on the presence of T cells [33], but our data also suggest that T cells are important to completely clear the virus from the lungs. Different studies have reported that adoptive transfer of cytotoxic CD8^+^ T cells helps to fully clear the persistent virus from the lungs of *Rag2^−/−^* mice during RSV [33, 44], respiratory poxvirus [50], and vaccinia virus infection [51]. Since the innate NK cell‐derived IFN‐γ in *Rag2^−/−^* mice cannot fully compensate for the CD8^+^ T cell‐derived IFN‐γ, one can argue that these two bursts of IFN‐γ may be functionally different, and further studies are needed to fully characterise the importance of IFN‐γ at different times post‐infection in both viral control and immunopathology.

A better understanding of the factors that enable the *Rag2^−/−^* mice to tolerate RSV without developing severe chronic inflammation in the lungs and airways will aid in the design of more effective treatments, prophylaxis, and vaccines to prevent complications from viral infections, particularly in immunocompromised individuals.

### 3.1 Data Limitations and Perspectives

The mechanisms controlling chronic viral infections in the lungs remain unclear. In this study, we demonstrate that NK cells, the major cytotoxic cells of the innate immune system, are dispensable for viral control during RSV infection in *Rag2* ^−/−^ mice on a C57BL/6 background. NK cells and ILC1 together comprise group 1 innate lymphoid cells [52, 53]. Although NK cells are over 40 times more abundant than ILC1 in *Rag2* ^−/−^ mice, both cell types express NK1.1 and rapidly produce IFN‐γ upon infection [54]. Therefore, our use of anti‐NK1.1 antibodies may have, in addition to depleting NK cells, also depleted ILC1, complicating the interpretation of their individual roles. A major limitation of our study was the inability to clearly distinguish NK cells from ILC1 via flow cytometry [55], preventing a definitive assessment of their respective contributions to IFN‐γ production and viral control. While ILC1 have been identified as the primary early source of IFN‐γ during influenza virus infection in mice [56], our data suggest that both NK cells and ILC1 are dispensable for RSV control in this model.

It is also important to note that we used *Rag2* ^−/−^ mice on a C57BL/6 background, which is more resistant to RSV than other strains like BALB/c [57]. Future studies should explore the long‐term role of NK cells in more severe models of RSV infection.

## 4 Materials and Methods

### 4.1 Ethics Approval Statement

All animal experiments were reviewed and approved by the Animal Welfare and Ethical Review Board (AWERB) within Imperial College London and approved by the UK Home Office in accordance with the Animals (Scientific Procedures) Act 1986.

### 4.2 Mice

C57BL/6J (Wt) mice were purchased from Charles River, UK. *Rag2* ^−/−^ mice were bred in‐house (obtained from Marina Botto, Imperial College London). All animals were bred and housed in specific pathogen‐free conditions. The mice were gender and age‐matched (8–12 weeks) in each experiment.

### 4.3 Virus and Infections

Plaque‐purified human RSV A2 (originally from ATCC) was grown in HEp‐2 cells (originally from ATCC) with DMEM (Sigma) supplemented with 2% fetal calf serum (FCS) (Sigma) and 2 mM L‐glutamine (Thermo Fisher). For infections, mice were transiently anesthetised with isoflurane and infected intranasally (i.n.) with 1.4 × 10^6^ focus‐forming units (FFU) of RSV or phosphate‐buffered saline (PBS) in 100 µl. The RSV titre was assessed in fresh lungs on the indicated days post‐RSV infection using an immunoplaque assay (optimised from [58]). Briefly, lung homogenates were titrated on HEp‐2 cell monolayers in six‐well, flat‐bottom plates and incubated for 2 h at 37°C 5% CO_2_. Then, pyruvate‐free DMEM (Sigma) containing 2% FCS was added, and the plates were further incubated for 44 h at 37°C 5% CO_2_, before being fixed with methanol (Sigma) and 2% hydrogen peroxide (Sigma). Biotin‐conjugated goat anti‐RSV antibody (Biogenesis) was added to the plates and incubated for 2 h at room temperature. Infected cells were detected using Extravidin‐peroxidase (Sigma‐Aldrich) and DAB substrate (diaminobenzidine tetrahydrochloride) (Sigma), enumerated by light microscopy, and used to calculate the viral titer as FFU.

### 4.4 Isolation of Cells from the Airway (BAL) and Lung

On the indicated day post‐RSV infection or PBS administration, mice were euthanised. The tracheae were exposed, and bronchoalveolar lavage (BAL) was performed by flushing the lungs three times with 1 ml PBS containing 0.5 mM EDTA (Life Technology, Paisley, UK). The fluid obtained was centrifuged at 6000 rpm for 5 min; supernatants were stored at −80°C for cytokine detection, and cellular pellets were treated with ACK to remove red blood cells. Cells were enumerated via Trypan Blue (Sigma) exclusion and used for flow cytometry staining. For lung cells, mice were perfused with 10 ml of PBS, and the lungs were excised. The top and middle‐right lobes were snap frozen in liquid nitrogen for RNA extraction, and the remaining three lobes were collected into C‐tubes (Miltenyi Biotech, UK) containing complete DMEM (cDMEM; DMEM supplemented with 10% FCS, 2 mM L‐glutamine, 100 U/ml penicillin, and 100 µg/ml streptomycin), 1 mg/ml Collagenase D (Roche, UK), and 30 µg/ml DNase I (Sigma Aldridge, UK) and processed with gentleMACS dissociator (Miltenyi Biotech) according to the manufacturer's protocol. Lungs were digested for 60 min at 37°C and further processed in the gentleMACS dissociator. Red blood cells were lysed using ACK buffer and then filtered using a 100 µm cell strainer. Cells were enumerated via Trypan Blue exclusion and used for flow cytometry staining.

### 4.5 NK Cell in Vivo Depletion

Mice were depleted of NK cells using the anti‐NK1.1 monoclonal antibody (PK136, eBioscience or AssayGenie) before and during RSV infection. 100 µg/mouse was given intranasally (i.n.) and 200 µg/mouse was given by intraperitoneal injection on day 1. 150 µg/mouse was then injected intraperitoneally every day for 2 days or 7 days. NK cell depletion was confirmed by flow cytometry of lung tissue for the presence of CD3^−^CD49b^+^ NK cells.

### 4.6 NK Cell Ex Vivo Stimulation and CD107a (LAMP1) Degranulation Assay‐

To detect intracellular cytokines from NK cells, isolated lung cells were stimulated with recombinant IL‐12 (5 ng/ml; PeproTech, UK) and IL‐18 (2.5 ng/ml; Biolegend, Cambridge, UK) [24], left unstimulated (medium) or received phorbol 12‐myristate 13‐acetate (PMA) (100 ng/ml) and ionomycin (300 ng/ml) (Abcam) and incubated for 1 h at 37°C 5% CO_2_. Golgi Plug (BD Biosciences) was then added 1 µl per 2.5 × 10^6^ cells according to the manufacturer's instructions and incubated for 3 additional hours. To measure the NK cell degranulation activity, anti‐mouse CD107a (1D4B, BV421, BioLegend) was added at 4 µg/ml to the cells during stimulation. Cells were then stained for flow cytometry analyses as described below.

### 4.7 Flow Cytometry

2.5 × 10^6^ cells isolated from the lung or BAL cells were incubated with purified rat IgG2b anti‐mouse CD16/CD32 receptor antibody (clone 93) for 20 min at 4°C (BioLegend). Cells were stained with fluorochrome‐conjugated antibodies (**Table** **S1**) for 30 min at 4°C before fixing the cells with fixation buffer (BioLegend). For intracellular staining, the fixed cells were incubated with permeabilisation buffer for 15 min, stained with fluorochrome‐conjugated antibodies against granzyme B (GrzmB) and IFN‐γ (XMG1.2) in the presence of purified rat IgG2b anti‐mouse CD16/CD32 receptor antibody in permeabilisation buffer (BioLegend) for 1 h. Samples were measured on a Becton Dickinson Fortessa. Data were analysed using FlowJo software (BD).

### 4.8 RNA Extraction and Quantitative RT‐PCR

Lung lobes were homogenized using a TissueLyser (Qiagen), and total RNA was extracted using a RNeasy Mini kit as described by the manufacturer (Qiagen), including the DNase step. RNA concentration was determined by NanoDrop (Thermo Scientific). Two micrograms of purified RNA was converted to cDNA using a High‐Capacity RNA‐to‐cDNA kit according to the manufacturer's protocol (Applied Biosystems). RT‐qPCR was performed with TaqMan Universal qPCR Master Mix (Qiagen) and the 7500 Fast Real‐Time PCR System (Applied Biosciences). For relative mRNA expression for *Il6*, *Ifna5, Viperin*, and *Mx1* (all from Applied Biosystems), the ΔCT was calculated for each target gene relative to *Gapdh* (encoding glyceraldehyde‐3‐phosphate dehydrogenase; Applied Biosystems) and expressed as 2^−ΔCT^. For exact quantification of mRNA for *Ifnb*, *Tnfa*, *Viperin*, and RSV *L gene*, copy numbers were calculated from the DNA standard curve and normalised to a housekeeping gene, *Gapdh* (Applied Biosystems), as previously described [11]. Analysis was performed using the 7500 Fast System SDS Software

### 4.9 Cytokine and Chemokine Detection

BAL supernatants were assessed for IFN‐γ and Granzyme B using an ELISA kit (R&D Systems, USA). Detection limits were 34 pg/ml for IFN‐γ, and 117 pg/ml for GrzmB. The FLUOstar OMEGA plate reader (BMG Labtech) was used at an absorbance of 450 nm. Data were analysed using the Omega/MARS data analysis software (BMG Labtech).

### 4.10 Statistical Analysis

For a simple two‐group comparison, an unpaired two‐tailed Student's *t*‐test was used. One ANOVA was used following Tukey's post hoc test for multiple comparisons. A value of *p* < 0.05 was considered significant for all tests. *p < 0.05; **p < 0.01; ***p < 0.001; ****p < 0.0001. Statistical analysis of data was performed using GraphPad Prism 10 (GraphPad Software Inc.).

## Author Contributions

Joy Nakawesi designed, performed, and analysed the experiments. Tammie Sow Tao Min performed specific experiments and reviewed the paper. Cecilia Johansson supervised the project and designed the experiments. Joy Nakawesi and Cecilia Johansson wrote the paper.

## Conflicts of Interest

The authors declare no conflicts of interest.

## Peer Review

The peer review history for this article is available at https://publons.com/publon/10.1002/eji.70045.

## Supporting information




**Supporting Figure 1**: eji70045‐sup‐0001‐SuppMat.pdf

## Data Availability

The data that support the findings of this study are available from the corresponding author upon reasonable request.
