# Supplementary material for: Natural Killer Cells Are Dispensable for Virus Control in Rag2−/− Mice During Primary RSV Infection
Source: Eur J Immunol. 2025 Sep 3;55(9):e70045. doi: 10.1002/eji.70045 (PMC12405976; doi:10.1002/eji.70045)
Supplement: Supplementary file 1 — Supporting Figure 1: eji70045‐sup‐0001‐SuppMat.pdf [file EJI-55-e70045-s001.pdf]

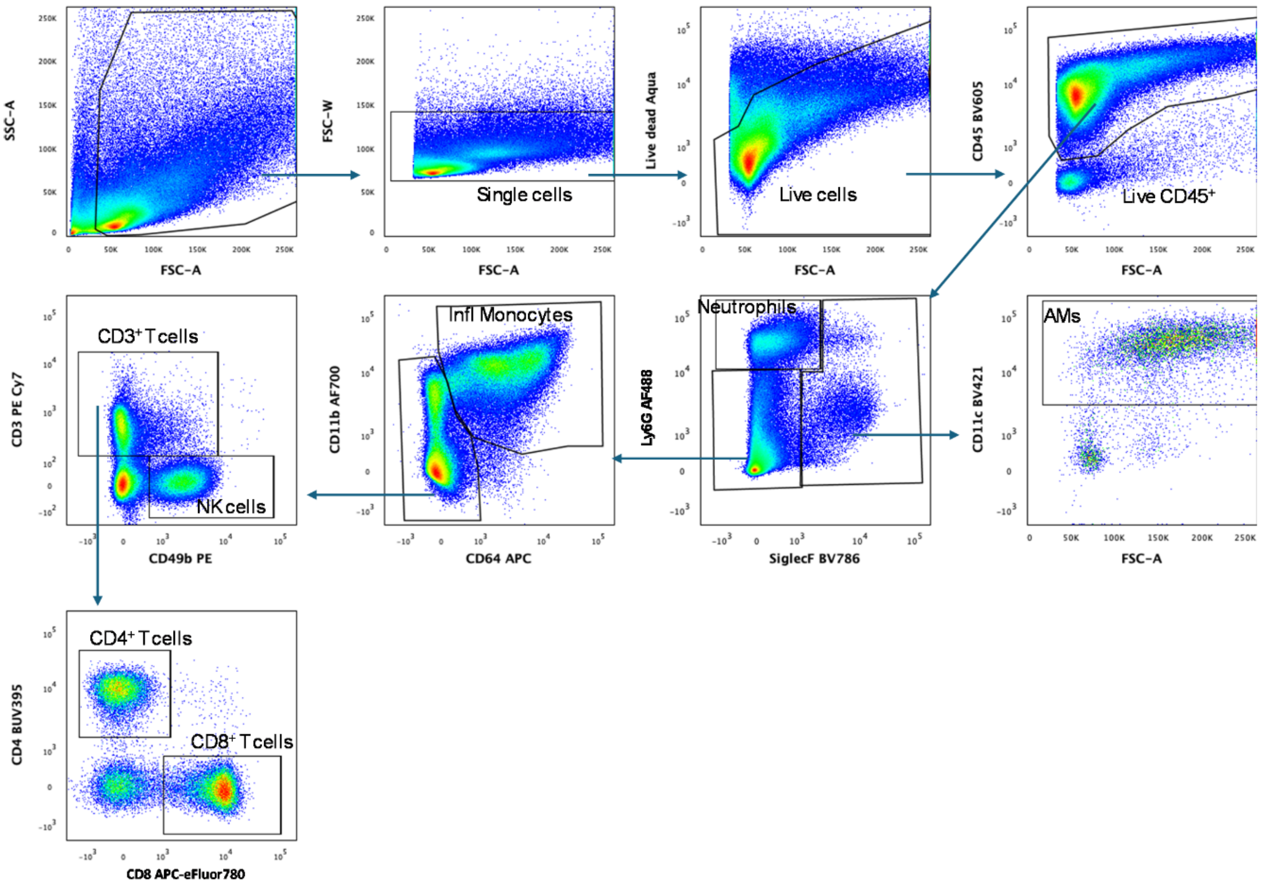


**Supplementary Figure 1. Gating strategy for identifying lung immune cells.**
Wt and *Rag2*$^{-/-}$ mice were mock (PBS) or RSV infected i.n.. Cells from the lungs and BAL were analysed at different time points after infection. Lung immune cells were obtained by collagenase digestion. Lung and BAL cells were stained for the indicated cell surface molecules. After excluding debris and gating on single, live, CD45$^{+}$ cells, the depicted gates were used to identify neutrophils (Ly6G$^{+}$SiglecF$^{-}$), alveolar macrophages (SiglecF$^{+}$CD11c$^{+}$), inflammatory monocytes (CD11b$^{+}$CD64$^{+}$), NK cells (CD49b$^{+}$), and T cells (CD3$^{+}$CD4$^{+}$ and CD3$^{+}$CD8$^{+}$). A representative lung sample for a Wt RSV-infected (day 2 p.i.) mouse is shown.

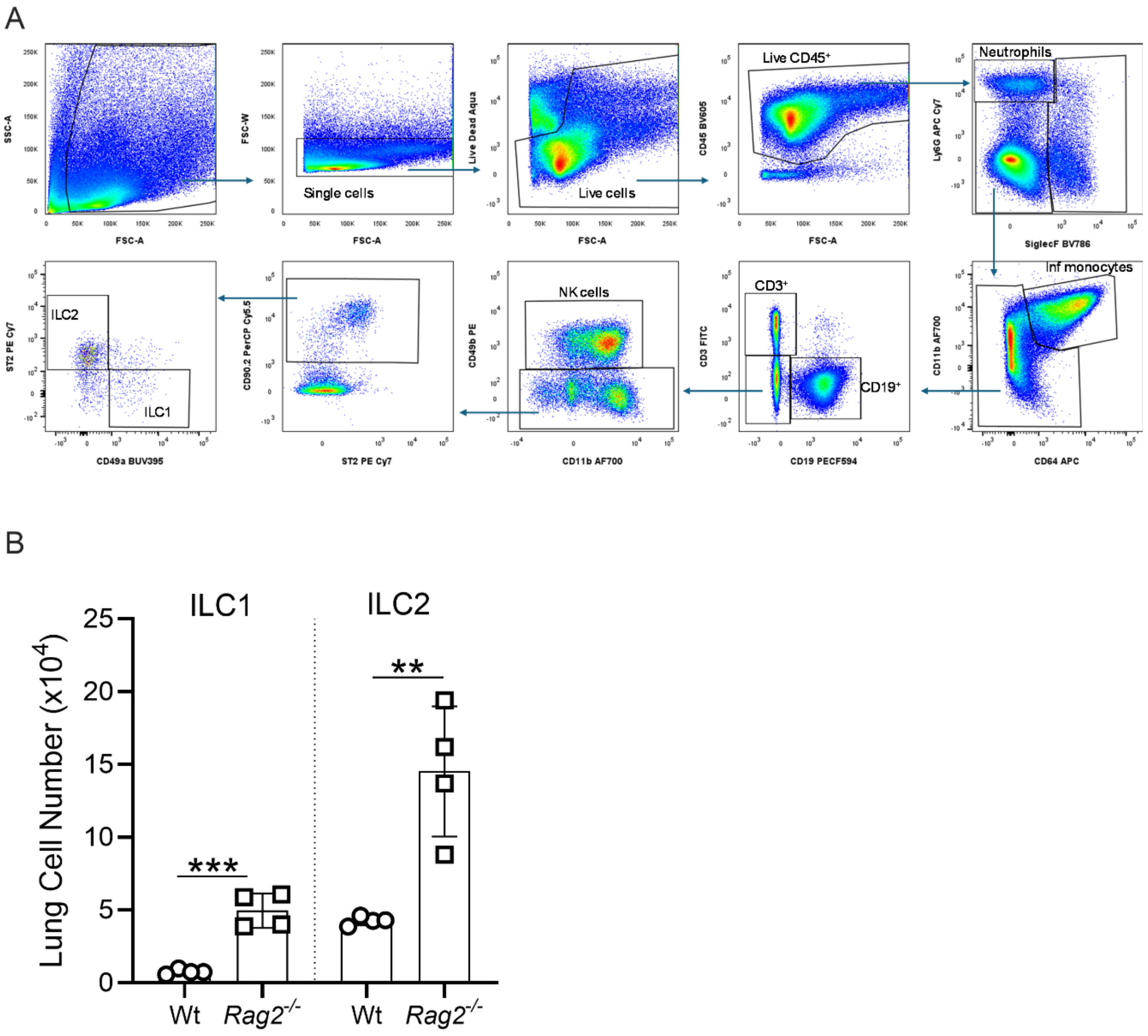


**Supplementary Figure 2. Innate lymphoid cells in the lungs of uninfected *Rag2$^{-/-}$* and Wt mice.** Wt and *Rag2$^{-/-}$* mice were mock (PBS) i.n., innate lymphoid cells 1 (ILC1) (Live, CD45$^{+}$Ly6G$^{-}$SigF$^{-}$CD64$^{-}$CD3$^{-}$CD19$^{-}$CD49b$^{-}$CD90.2$^{+}$ST2$^{-}$CD49a$^{+}$) and innate lymphoid cells 2 (ILC2) (Live ,CD45$^{+}$Ly6G$^{-}$SigF$^{-}$CD64$^{-}$CD11b$^{-}$CD3$^{-}$CD19$^{-}$CD49b$^{-}$CD90.2$^{+}$CD49a$^{-}$ST2$^{+}$) were analysed on day 2 p.i.. (**A**) Representative flow cytometry plots showing the gating strategy for identifying group ILC1 and ILC2 in the lungs. (**B**) The total number of ILC1 and ILC2 in uninfected mice. Data are presented as the mean±SEM of Wt n=4 mice and *Rag2$^{-/-}$* n=4 mice from one experiment Each symbol represents an individual mouse. Statistical significance was determined by Student's *t test*. **p<0.01, ***p<0.001.

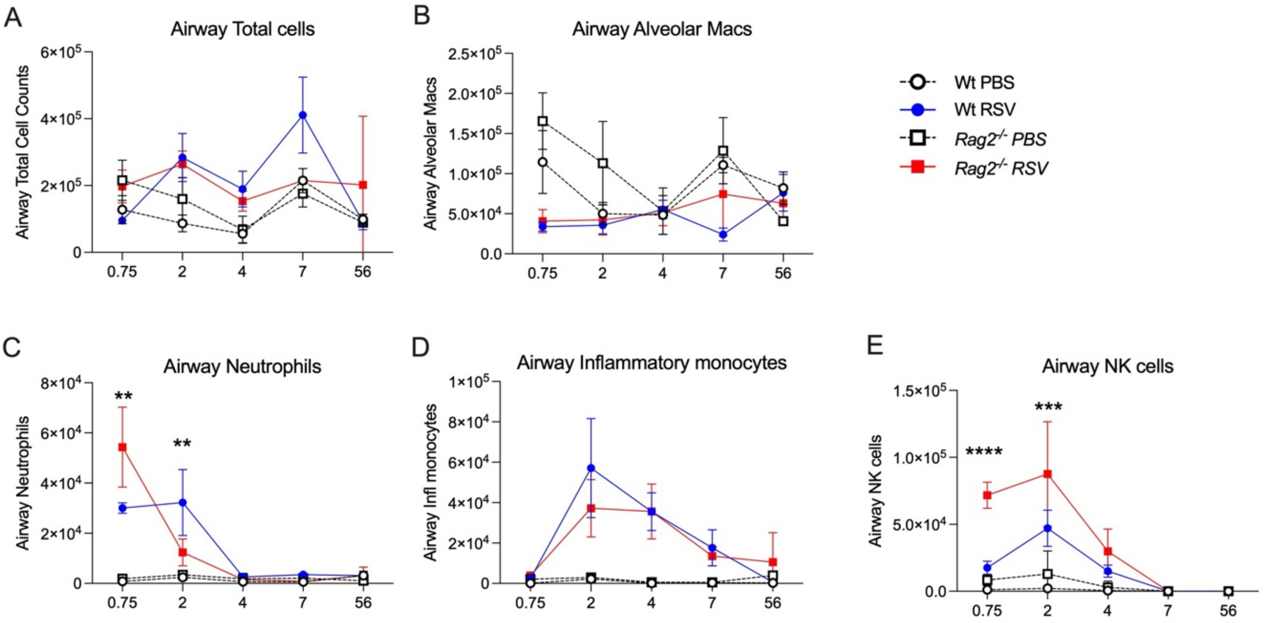


**Supplementary Figure 3. Innate immune cells in the airways after RSV infection in *Rag2$^{-/-}$* and Wt mice.**

Wt and *Rag2$^{-/-}$* mice were mock (PBS) or RSV infected i.n. and the airway (BAL) cells were analysed at different time points after infection. (**A**) Total cells in the airways, (**B**) alveolar macrophages, (**C**) neutrophils, (**D**) inflammatory monocytes and (**E**) NK cells. Data are presented as the mean±SEM of n=3-4 mice/group from one experiment (day 0.75), n=6-8 mice/group pooled from two independent experiments (day 2 and 4), n=3-6 mice/group from one experiment (day 7 and 56). Statistical significance was determined by one-way ANOVA with Tukey's post hoc test per time point, and only significance between RSV groups is shown. **p<0.01, ***p<0.001, ****p<0.0001. Symbols: open circle: Wt PBS; open square: *Rag2$^{-/-}$* PBS; blue circle: Wt RSV; red square: *Rag2$^{-/-}$* RSV.

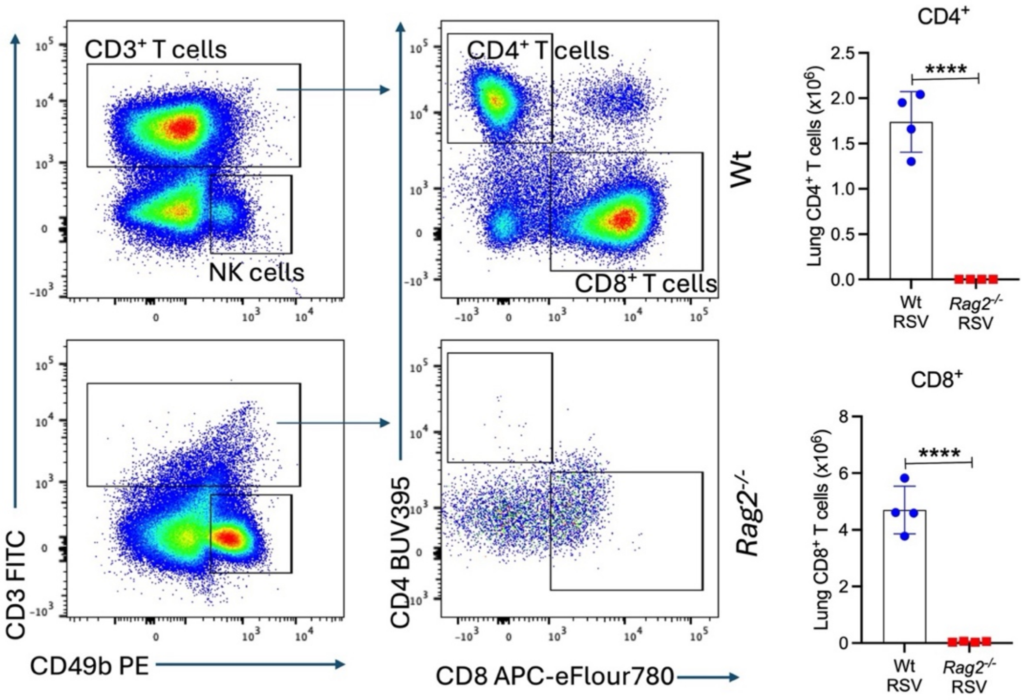


**Supplementary Figure 4. T cells in the lungs of *Rag2^-/-^* and Wt mice after RSV infection.**
Wt and *Rag2$^{-/-}$* mice were mock (PBS) or RSV infected i.n. and T cells ($CD3^+CD4^+$ and $CD3^+CD8^+$) were analysed in the lungs on day 7 p.i.. Data are presented as the mean±SEM of n=4 Wt and n=4 *Rag2$^{-/-}$* individual mice from one experiment representative of two independent experiments. Statistical significance was determined by Student's *t test*. ****p<0.0001. Symbols: blue circle: Wt RSV; red square: *Rag2$^{-/-}$* RSV. Each symbol represents an individual mouse.

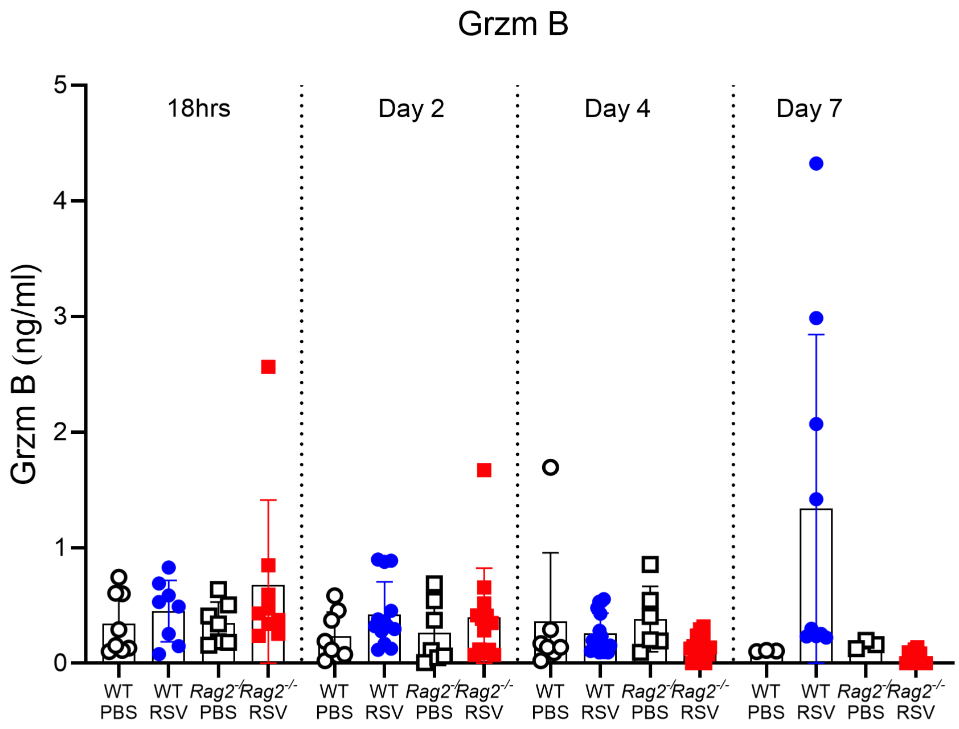


**Supplementary Figure 5. Granzyme B levels after RSV infection in *Rag2$^{-/-}$* and Wt mice.**
Wt and *Rag2$^{-/-}$* mice were mock (PBS) or RSV infected i.n. and the BAL fluid was analysed for granzyme B levels by ELISA at day 0.75, 2, 4 and 7 p.i.. Data are presented as the mean±SEM of n=7-8 mice/group pooled from 2 independent experiments (day 0.75), n=7-8 for PBS groups and n=12-14 for RSV-infected mice pooled from 2 or 3 independent experiments respectively (day 2 and 4) and n=3 for PBS groups (from one experiment) and n=9-10 for RSV-infected mice pooled from 2 independent experiments (day 7). No statistical significance was detected using one-way ANOVA with Tukey's post hoc test per time point. Symbols: open circle: Wt PBS; open square: *Rag2$^{-/-}$* PBS; blue circle: Wt RSV; red square: *Rag2$^{-/-}$* RSV. Each symbol represents an individual mouse.

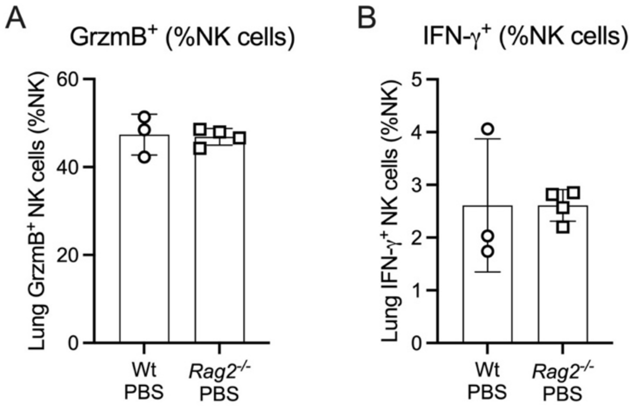


**Supplementary Figure 6. NK cell functionality in non-infected Wt and *Rag2$^{-/-}$* mice.**
Wt and *Rag2$^{-/-}$* mice were given PBS i.n. and after 2 days, cells from the lungs were stimulated *ex vivo* with IL-12 and IL-18 and subsequently stained for intracellular (**A**) granzyme B and (**B**) IFN-γ. The frequency of cells is presented as the mean±SEM of Wt n=3 mice and *Rag2$^{-/-}$* n=4 mice from one experiment. Each symbol represents an individual mouse. No statistical significance was detected using Student's *t test*.

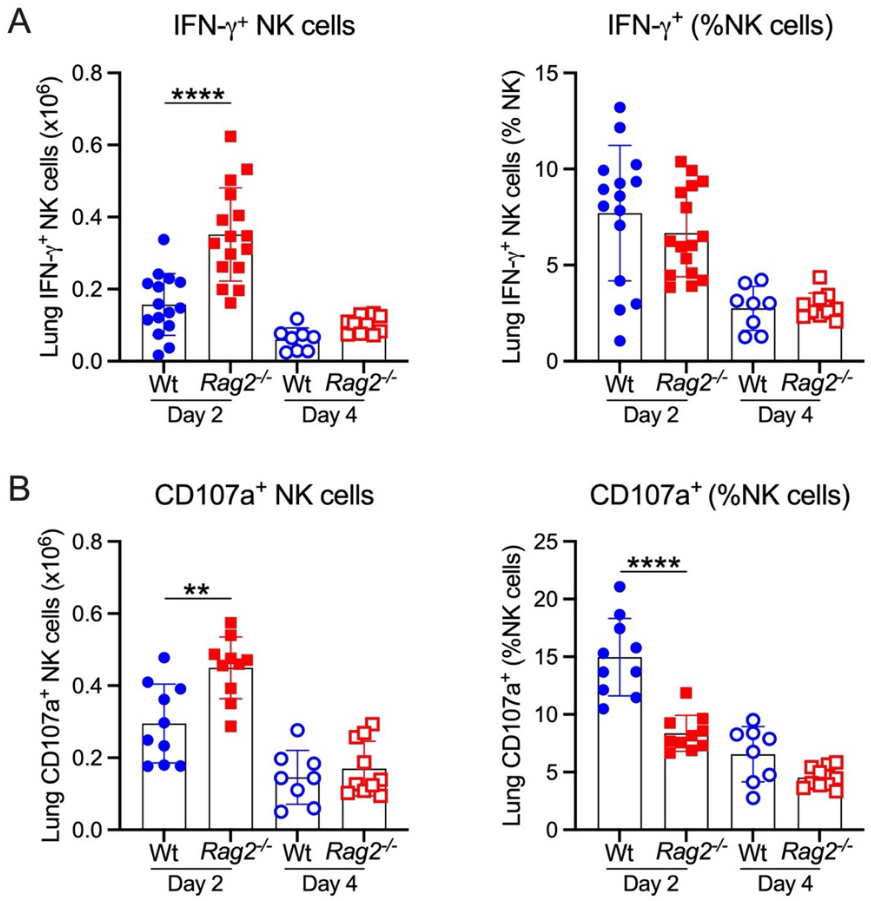


**Supplementary Figure 7. NK cell functionality on day 2 and day 4 post RSV infection in Wt and *Rag2$^{-/-}$* mice.**

Wt and *Rag2$^{-/-}$* mice were RSV infected i.n.. On days 2 and 4 p.i., lung cells were stimulated *ex vivo* with IL-12 and IL-18. The cells were subsequently stained for surface CD107a and intracellular IFN-γ. NK cells in the lungs were analysed for (**A**) intracellular IFN-γ and (**B**) surface CD107a. Data are presented as the mean±SEM of (**A**) Wt n=15 and *Rag2$^{-/-}$* n=16 pooled from 3 independent experiments (day 2), and Wt n=8 and *Rag2$^{-/-}$* n=10 pooled from 2 independent experiments (day 4). (**B**) Wt n=8-10 and *Rag2$^{-/-}$* n=10 pooled from 2 independent experiments (day 2 and 4). Each symbol represents an individual mouse. Statistical significance was determined by Student's *t* test at each time point. **p<0.01, ****p<0.0001. Blue symbols: Wt RSV; red symbols: *Rag2$^{-/-}$* RSV.

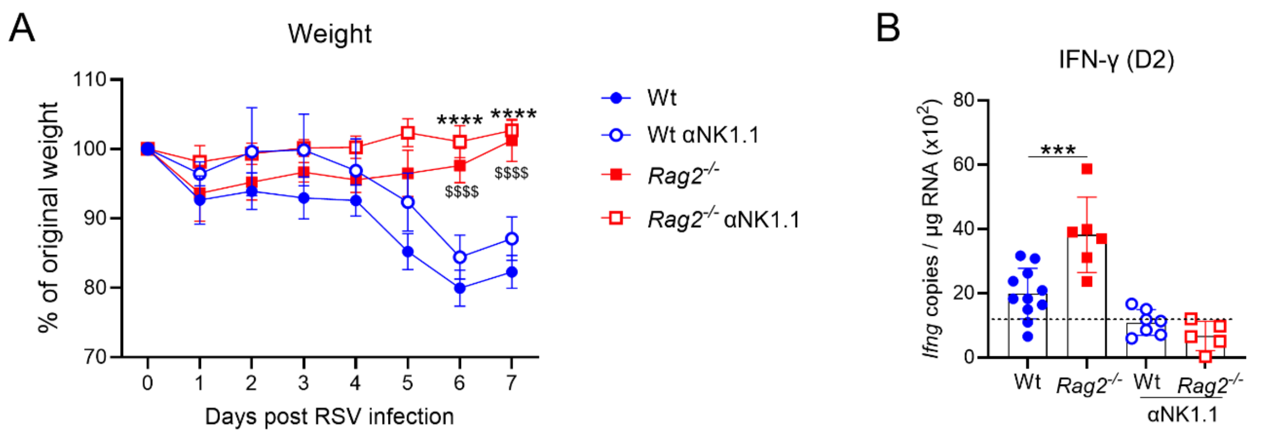


**Supplementary Figure 8. NK cells do not contribute to RSV disease severity as measured by weight loss.**

Wt and *Rag2*$^{-/-}$ mice were RSV infected i.n., some RSV-infected mice were depleted of NK cells using the anti-NK1.1 monoclonal antibody. (A) Weight loss is shown as the percentage of the original weight. (B) mRNA expression of *Ifng* determined by RT-qPCR in the lung tissue on day 2 p.i.. Data are presented as the mean±SEM of (A) Wt n=4 mice/group and *Rag2*$^{-/-}$ n=5 mice/group from one experiment. (**B**) Wt n=7-11 mice/group and *Rag2*$^{-/}$ n=5-6 mice/group pooled from 2 independent experiments. Each symbol represents an individual mouse. Statistical significance was determined by (**B**) one-way ANOVA with Tukey's post hoc test. (**A**) No statistical significance was found using a one-way ANOVA with Tukey's post hoc test per time point between RSV-infected Wt and Wt NK cell-depleted mice or RSV-infected *Rag2*$^{-/-}$ and *Rag2*$^{-/-}$ NK cell-depleted mice. Statistical significance is shown as stars (*) between RSV-infected Wt and *Rag2*$^{-/-}$ mice and dollars ($) between RSV-infected NK cell-depleted Wt and *Rag2*$^{-/-}$ mice. *** p<0.001, ****, $$$$ p<0.0001. Symbols: open circle: Wt RSV-αNK1.1; open square: *Rag2*$^{-/-}$ RSV-αNK1.1; blue circle: Wt RSV; red square: *Rag2*$^{-/-}$ RSV.

**Supplementary Table 1**: Antibodies used for flow cytometry

| **Antibody targeting:** | **Company** | **Clone** | **Catalogue number** |
|---|---|---|---|
| CD16/CD32 | BioLegend | 93 | 101302 |
| CD45 AF488 | BioLegend | 30-F11 | 103122 |
| CD45 BV605 | BioLegend | 30-F11 | 103140 |
| CD11b AF700 | Invitrogen | M1/70 | 56-0112-82 |
| CD11c BV421 | BD Biosciences | HL3 | 562782 |
| CD4 BUV395 | BD Biosciences | GK1.5 | 563790 |
| CD3 FITC | BioLegend | 145-2C11 | 100306 |
| CD3 PE Cy7 | BD Biosciences | 145-2C11 | 552774 |
| CD8 APC efluor780 | Invitrogen | 53-6.7 | 47-0081-82 |
| CD64 APC | BioLegend | X54-5/7.1 | 139306 |
| SiglecF BV786 | BD Biosciences | E50-2440 | 740956 |
| Ly6G PE-Cy7 | BioLegend | 1A8 | 127618 |
| Ly6G AF488 | BioLegend | 1A8 | 127626 |
| CD49b PE | BioLegend | DX5 | 108908 |
| Fixable live-dead Aqua dye | Invitrogen | | L34966 |
| CD107a BV421 | BD Biosciences | 1D4B | 564336 |
| IFN-γ BV711 | BD Biosciences | XMG1.2 | 564336 |
| IFN-γ BV605 | BD Biosciences | XMG1.2 | 505839 |
| Granzyme B PECF594 | BD Biosciences | GB11 | 562462 |
| CD90.2 (Thy1.2) PerCP Cy5.5 | BioLegend | 30-H12 | 105337 |
| IL-33Ra (ST2) PE Cy7 | BioLegend | DIH4 | 146609 |

LSR equipped with 20mW 355nm, 50mW 405nm, 50mW 488nm, 50mW 561nm, and 20mW 633nm lasers and an ND1.0 filter in front of the FSC photodiode. For acquisition, PMT voltages were set after CST standardised checks to maximise data precision and 250,000 single, live $CD45^+$ events were recorded.
